# Changes of Peripheral Blood Lymphocyte Subtypes in Patients with End Stage Cancer Administered Localized Radiotherapy and Bojungikki-Tang

**DOI:** 10.1155/2014/207613

**Published:** 2014-02-20

**Authors:** A-Jin Lee, Ho Jun Lee, Jong-Dae Kim, Hyun-Jung Jung, Sung Hwa Bae, Hun Mo Ryoo, Sang-Gyung Kim

**Affiliations:** ^1^Department of Laboratory Medicine, School of Medicine, Catholic University of Daegu, Daegu 705-718, Republic of Korea; ^2^Department of Radiotherapy, School of Medicine, Catholic University of Daegu, Daegu 705-718, Republic of Korea; ^3^Department of Internal Medicine, College of Korean Medicine, DaeguaHany University, Daegu 712-715, Republic of Korea; ^4^Department of Diagnostics, College of Korean Medicine, DaeguaHany University, Daegu 712-715, Republic of Korea; ^5^Division of Hematology-Oncology, Department of Internal Medicine, School of Medicine, Catholic University of Daegu, Daegu 705-718, Republic of Korea; ^6^Comprehensive Integrated Medicine Institute, Daegu 705-825, Republic of Korea

## Abstract

Localized radiotherapy (RT) can cause immune dysfunction. Bojungikki-tang is known to restore immune function. We investigated the absolute counts and percentages of peripheral blood (PB) lymphocyte subtypes in end stage cancer patients before and after RT and after oral administration of Bojungikki-tang water extract (BJITE) and to evaluate the changes mediated by RT and BJITE. Absolute counts and percentages of lymphocyte and lymphocyte subsets were determined in whole blood using the TetraONE System (Beckman Coulter, USA). Flow cytometry results were compared before and after RT and after administration of BJITE. Absolute numbers of CD3+, CD4+, and CD8+ T cells and CD19+ B cells decreased significantly after RT (*P* < 0.05). Absolute numbers of CD3-CD56+ cells did not change in both groups. No significant differences were observed in the absolute counts of lymphocyte subtypes before and after administration of BJITE or vitamin group. When BJITE group was compared with vitamin group, absolute numbers of CD19+ B cells increased. RT-induced decrease in T cells and B cells in PB suggests that immune deterioration occurs after RT. Administration of BJITE might be effective in the restoration of number of B cells.

## 1. Introduction 

Radiotherapy (RT) can be used to control symptoms of patients with end stage cancer. RT with palliative intent is administered in approximately one-half of cancer patients [1]. The purposes of palliative RT are to decrease symptoms, including pain, bleeding, and obstruction, and to improve the patient's quality of life [1, 2]. However, there is an adverse effect of palliative RT. It has been demonstrated that localized radiation, even if administered to limited target volume, causes immune dysfunction [3–5].

Bojungikki-tang (Hochuekkito in Japanese and Bu-zhong-yi-qi-tang in Chinese) is a traditional herbal formula used in Korea, Japan, and China and it is composed of 10 species of medicinal plants. It has been traditionally used to improve severe weakness in Asian countries. Recent studies have demonstrated that Bojungikki-tang water extract (BJITE) has effect on restoring immune function [6] and inducing an increased protection against microbial agents [7]. It is useful not only for an enhancement of natural killer (NK) activity [8] but also for restoration of antitumor T cell response from stress-induced suppression [9]. Bojungikki-tang is known to have protective effect of intestine and hematopoietic organs against radiation damage [10]. However, the immunological response of Bojungikki-tang in patients administrated RT has not yet been determined.

The aims of the present study were to determine the absolute counts and percentages of peripheral blood (PB) lymphocyte subtypes in end stage cancer patients before and after RT and after oral administration of Bojungikki-tang using single-platform technology and to evaluate the changes mediated by RT and Bojungikki-tang.

## 2. Methods and Materials

### 2.1. Patients

Thirteen patients were enrolled in this single center, randomised controlled study. Patients meeting the following criteria were included: age 40 years or older; ECOG performance score 0–2; end stage cancer; selected for palliative RT. Exclusion criteria were as follows: patients who have been treated with operation, chemotherapy, or curative radiotherapy in the 2 months prior to randomisation; patients with severe hepatic or renal dysfunction (AST > 80 IU/L, ALT > 80 IU/L, BUN > 50 mg/dL, and creatinine > 3.4 mg/dL); subjects with a history or hypersensitivity to functional foods; women who are pregnant or nursing; patients who are of child bearing age and are not willing to use contraception; patients who have had major surgery in the 3 months prior to randomization; patients with neuropsychiatric disease; patients who have had cardiovascular or cerebrovascular disease during the previous 6 months; patients who have taken drug in the 3 months prior to randomization. All patients had to provide written informed consent before registration and the trial protocol was approved by the Institutional Review Board of Daegu Catholic University Medical Center, Korea.

Eligible patients were randomly assigned (1 : 1) to palliative RT with Bojungikki-tang (BJITE group) or palliative RT with vitamin (vitamin group).  Table 1 shows the patients' clinical characteristics. All patients except one suffered from metastatic tumor.

### 2.2. Procedures

All patients were treated with external RT using a linear accelerator with 6 and 10MV (Varian 21EX linear accelerator equipped with standard multileaf collimators) for 2 to 5 weeks. Fractions of 1.5–3.0 Gy were delivered 5 days/wk for a total dose of 30–50 Gy. After completing RT, BJITE group was orally administered 9.0 g of Bojungikki-tang everyday (4.5 g × 2) throughout the 4-week period. Bojungikki-tang was manufactured as a spray-dried powder of hot water extract obtained from 10 medical plants composed of Ginseng radix, Atractylodis rhizoma, Astragali radix, Angelicae radix, Aurantii nobilis pericarpium, Zizyphi fructus, Bupleuri radix, Glycyrrhizae radix, Zingiberis rhizoma, and *Cimicifugae* rhizoma. Placebo (vitamin) group was taken as non-Bojungikki-tang control. Vitamin tablet was Co-Q ten vitaalbu tab (Ilyang Pharmaceutical Co., Korea) containing 280.9 mg ascorbic acid, 2.5 mg cupric oxide, 5 mg 0.1% cyanocobalamin, 0.47 mg dried ergocalciferol powder, 20 mg dried retinol acetate powder, 60.8 mg ferrous fumarate, 41.4 mg magnesium oxide, 1.58 mg manganese dioxide, 100 mg nicotinamide, 15 mg pyridoxine hydrochloride, 30 mg riboflavin, 30 mg thiamine nitrate, 60 mg tocopherol acetate 50%, 10 mg ubidecarenone, and 1.87 mg zinc oxide. Vitamin was given orally daily throughout the 4-week period. The administration of any drugs known to affect the host immunity was avoided during this period.

### 2.3. Blood Collection

Whole blood samples were collected one day before starting RT (Time 0), one day after completing RT (Time 1), and four weeks after oral administration of BJITE or vitamin (Time 2) in sterile EDTA vacutainers.

### 2.4. Flow Cytometric Immunophenotyping Using Fluorochrome-Conjugated Antibodies

The percentages and absolute lymphocyte counts were determined in whole blood using a standard single-platform technique, the TetraONE System (Beckman Coulter, Miami, USA), based on four-color flow cytometry in the presence of counting beads. The following combinations were used during immunofluorescence analysis: tube1, anti-CD45-FITC/anti-CD4-PE/anti-CD8-ECD/anti-CD3-PC5, and tube 2, anti-CD45-FITC/anti-CD56-PE/anti-CD19-ECD/anti-CD3-PC5. For each specimen, 100 *μ*L of EDTA-anticoagulated blood was added to 10 *μ*L of tetraCHROME reagent containing the four-antibody-fluorochrome combinations and incubated for 20 min at room temperature in a dark room. Specimens were then lysed using the ImmunoPrep Reagent System at the Coulter Multi-Q-Prep Workstation. Immediately prior to analysis, 100 *μ*L of Flow-Count Fluorospheres (Beckman Coulter) was added to each tube, and the beads were counted along with cells. The sample acquisition and flow cytometric immunophenotypic analysis were performed on the FCM, Cytomics (Beckman Coulter), with a fully automated software-reagent combination. The identification of lymphocytes by expression of bright CD45 and low side scatter signals was followed by the identification of T cell subtypes based on the expression of CD3, CD4, and CD8. B cell subsets and natural killer (NK) cell subsets were based on the expression of CD19 and CD3-CD56+, respectively. The absolute count of cells per microliter was obtained by calculating the number of cells counted × concentration of beads/number of beads counted.

### 2.5. Statistical Analysis

The data are presented as mean and SD. Mann-Whitney *U* test and the chi-square test were used when appropriate to compare distribution of individual variable between groups. Wilcoxon's signed rank sum test was used to compare change of absolute lymphocyte count and lymphocyte subset. The two-sided *P* values were considered significant at *P* < 0.05. SPSS software version 19.0 (SPSS Inc., Chicago, IL) was used for statistical analysis.

## 3. Results

### 3.1. Percentages and Counts of Total Lymphocytes and Lymphocyte Subsets after Radiotherapy

**Figure 1:**
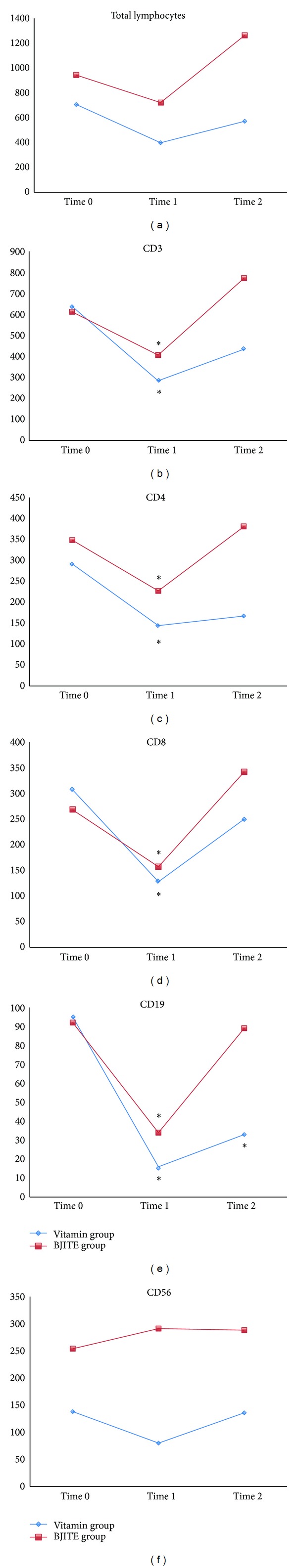
Lymphocyte subset counts during RT in vitamin group versus Bojungikki-tang extract (BJITE) group.

Using the single-platform technology, we initially compared both the percentages and absolute counts of lymphocyte subsets in all patients. Absolute counts and percentages of lymphocytes and lymphocyte subsets after RT in the BJITE group (*n* = 7) versus vitamin group (*n* = 6) are shown in Tables 2 and 3, respectively. There were no significant changes in total lymphocyte counts or percentages of lymphocytes during period of time in both groups. No significant differences were observed in the percentages of lymphocyte subtypes. However, absolute numbers of CD3+, CD4+, and CD8+ T cells and CD19+ B cells decreased significantly after RT (*P* < 0.05) (Figure 1). Absolute numbers of CD3-CD56+ cells did not change in both groups.

### 3.2. Effects of BJITE on Total Lymphocytes and Lymphocyte Subsets

Absolute counts and percentages of lymphocytes and lymphocyte subsets after administration of BJITE or vitamin are shown in Tables 2 and 3, respectively. After administration of BJITE, there were no differences in total lymphocyte number and all lymphocyte subsets compared with baseline data. No differences were found, before and after administration of BJITE or vitamin group, in the CD3+, CD4+, and CD8+ T cells as well in of CD56+ cells. However, there were significant differences in CD19+ B cell counts and percentages of CD19 in vitamin group. In vitamin group, the percentages and absolute counts of CD19 cells did not increase compared with baseline status and remained the decreased status.

## 4. Discussion

Bojungikki-tang is known to restore immune functions and to improve anti-tumor activity [11–13]. It is usually indicated for patients with general weakness and anemia. Palliative RT relieves clinical symptoms in advanced cancer patients but it can also cause immunological changes. In the present study, we have investigated the percentages and absolute counts of PB lymphocytes and lymphocyte subtypes in patients with end stage cancer administered localized radiotherapy and Bojungikki-tang.

In our study, total lymphocyte in counts and percentages of lymphocytes did not change after palliative RT in both groups. This is contrast to the results of the earlier study of radiation-induced lymphocytopenia [3–5, 14]. This is probably due to differences of radiation dose and interval.

Absolute counts of T cells and B cells declined after localized RT. Absolute counts of NK cells were not affected by RT. Local radiation at therapeutic doses always triggered some activation of the innate and adaptive immune system [15]. Results of present study correspond well with those of the earlier study, which reported that naive T cells and B cells are highly radiosensitive [16, 17]. No significant differences were observed in the percentages of lymphocyte subtypes in all patients. These findings differ from the results of the earlier study, which reported that, unlike absolute numbers, the percentages of all analyzed lymphocyte subsets of cervical cancer patients significantly elevated after RT [18]. Safwat et al. [14] reported that localized RT is associated with a significant increase in the percentage of CD4+ T cells and a significant reduction of the absolute number of lymphocyte subsets in patients with non-Hodgkin's lymphoma. This result is similar to our result except for the increase of CD4+ T cell percentages.

Absolute counts of lymphocyte subpopulations in PB have been traditionally measured by dual-platform technologies, which were standard, widely used methodologies. These methods couple percentages of positive cell subsets determined by flow cytometry with the absolute lymphocyte count obtained by automated hematology analyzers. However, they may be responsible for substantial differences in absolute lymphocyte counts determined by different analyzers [19]. Recently, single-platform technologies were developed and are performed entirely on flow cytometry. These methods have significantly improved the assay precision and accuracy and agreement of results between laboratories [19, 20]. Using the single-platform flow cytometry, it was possible to independently analyze the percentages and absolute counts of lymphocyte subsets. In this study, absolute counts of lymphocyte subpopulations decreased significantly after RT, although the percentages of lymphocyte subsets did not change. The values of percentages did not consider total WBC count, which might be changed in cancer patients, particularly after administration of RT. Therefore, it may be useful to determine absolute counts, not percentages, of lymphocyte subsets for exactly reflecting immune status of patients.

Absolute counts or percentages of lymphocytes from BJITE or vitamin group showed no significant differences before and after administration of BJITE or vitamin. Another study also reported that the total number of circulating lymphocytes; CD3+, CD4+, and CD8+ T cells; and CD20+ B cells remained unchanged [21].

Interestingly, CD19+ B cells increased significantly after administration of BJITE. These findings are in contrast to the results of the earlier study, which reported that BJITE is remarkably effective in the restoration of number of T cells and NK cells [8]. Bojungikki-tang is known to have beneficial effects on anti-tumor activity [9] or NK cell activity [21]. In this study, measurement of NK cell activity was not performed. The administration of Bojungikki-tang polysaccharide fraction was associated with elevated expression levels of CD19/CD40 specific for pre-B cells [22].

## 5. Conclusions

In conclusion, RT-induced decrease in helper T cells, cytotoxic T cells, and B cells in PB suggests that immune deterioration occurs after RT. Administration of BJITE might be effective in the restoration of number of B cells.

## Figures and Tables

**Table 1:** Patient clinical characteristics.

	Vitamin group (*N* = 6)	BJITE group (*N* = 7)
Age (range)	64 ± 6 (54–70)	66 ± 6 (59–76)
Sex (f/m)	3/3	3/4
Primary site	Esophagus (1)	Lung (3)
	Pyriform sinus (1)	Rectum (2)
	Breast (1)	Common bile duct (1)
	Liver (1)	Gastrointestine (1)
	Ovary (1)	
	Uterus (1)	
Metastatic tumor	Bone (1)	Bone (5)
	Lymph node (3)	Brain (1)
	Chest (1)	Liver (1)

**Table 2:** Lymphocyte subset counts (cells/*μ*L) in the peripheral blood of end stage cancer patients during study period.

Cell type	Surface markers	Time 0		Time 1		Time 2	
		BJITE group	Vitamin group	BJITE group	Vitamin group	BJITE group	Vitamin group
Total lymphocytes		944 ± 287	707 ± 328	722 ± 355	399 ± 165	1263 ± 567	572 ± 261
T lymphocytes	CD3^+^	612 ± 183	638 ± 401	406 ± 240*	284 ± 84*	772 ± 331	434 ± 193
Helper	CD4^+^	348 ± 189	291 ± 106	227 ± 180*	144 ± 47*	381 ± 233	167 ± 64
Cytotoxic	CD8^+^	268 ± 116	307 ± 300	157 ± 98*	128 ± 58*	342 ± 169	250 ± 145
	CD4^+^/CD8^+^ ratio	1.58 ± 1.15	1.33 ± 0.71	1.71 ± 1.04	1.51 ± 1.08	1.36 ± 1.05	0.81 ± 0.34
B lymphocytes	CD19^+^	92 ± 63	95 ± 66	34 ± 27*	16 ± 19*	89 ± 68	33 ± 28*
NK cells	CD56^+^	254 ± 158	138 ± 40	291 ± 322	80 ± 95	288 ± 257	136 ± 128

*P value < 0.05 was considered statistically significant (Time 0 versus Time 1 and Time 0 versus Time 2 by Wilcoxon's signed rank sum test).

**Table 3:** The percentages of lymphocyte subsets in the peripheral blood of end stage cancer patients during study period.

Cell type	Surface markers	Time 0		Time 1		Time 2	
		BJITE group	Vitamin group	BJITE group	Vitamin group	BJITE group	Vitamin group
Total lymphocytes		12.2 ± 3.8	11.6 ± 2.8	10.4 ± 5.1	10.5 ± 3.3	17.3 ± 8.7	14.9 ± 7.5
T lymphocytes	CD3^+^	61.2 ± 11.3	70.2 ± 6.4	62.2 ± 19.7	76.3 ± 10.6	64.8 ± 13.1	71.4 ± 9.5
Helper	CD4^+^	33.0 ± 14.1	35.1 ± 7.6	35.9 ± 18.1	39.4 ± 11.9	31.3 ± 7.7	28.8 ± 7.0
Cytotoxic	CD8^+^	28.4 ± 13.7	31.1 ± 11.6	23.3 ± 7.0	34.3 ± 17.0	30.6 ± 13.8	40.3 ± 15.3
B lymphocytes	CD19^+^	8.8 ± 6.0	10.2 ± 6.1	5.0 ± 2.9	4.0 ± 4.4*	7.2 ± 3.8	6.1 ± 5.0*
NK cells	CD56^+^	25.7 ± 13.9	17.5 ± 7.8	30.1 ± 20.0	16.7 ± 11.1	24.8 ± 15.7	20.7 ± 11.2

*P value < 0.05 was considered statistically significant (Time 0 versus Time 1 and Time 0 versus Time 2 by Wilcoxon's signed rank sum test).
